# Implicit Motor Learning Under Anodal or Cathodal tDCS During fMRI Induces Partially Distinct Network Responses

**DOI:** 10.1111/ejn.70053

**Published:** 2025-03-12

**Authors:** Farsin Hamzei, Alexander Ritter, Daniel Güllmar

**Affiliations:** ^1^ Section of Neurological Rehabilitation, Clinic of Neurology University Hospital Jena Jena Germany; ^2^ Department of Neurology Moritz Klinik Bad Klosterlausnitz Bad Klosterlausnitz Germany; ^3^ Medical Physics Group, Department of Radiology University Hospital Jena Jena Germany

**Keywords:** anodal transcranial direct current stimulation, cathodal transcranial direct current stimulation, finger tapping, implicit motor learning, network plasticity, serial reaction time task (SRTT)

## Abstract

How anodal transcranial direct current stimulation (atDCS) and cathodal tDCS (ctDCS) affect brain networks is still unclear. Previous fMRI studies have yielded controversial results regarding the effects of atDCS and ctDCS on fMRI activation. The present study hypothesizes that the choice of fMRI paradigm may be a contributing factor to this divergence. Therefore, the present study employed two distinct fMRI paradigms, characterized by varying degrees of complexity: finger tapping as a simple fMRI paradigm and an implicit serial reaction time task (SRTT) as a more challenging paradigm. Seventy‐five healthy subjects were randomized to receive either atDCS, ctDCS, or sham stimulation during fMRI. The main effects of the blood oxygenation level–dependent (BOLD) signal were contrasted between groups. SRTT, but not FT, was capable of eliciting differences in modulatory effects on the network between groups. Analysis of functional connectivity between ROIs showed that atDCS and ctDCS shared common and distinct SRTT networks. Correlations between BOLD signal (in ROIs) and the reaction time (RT) recorded during fMRI showed that in the atDCS group, faster RT was associated with higher BOLD signal in the most ROIs, while in the ctDCS group, faster RT was mostly associated with lower BOLD signal activity. The sham group exhibited a combination of these associations. We suggest that atDCS accelerates RT by “pushing” the network, while the network response under ctDCS was a “compensatory” response. The polarity of tDCS differentially modulated the adaptive plasticity of remotely connected regions, based on the concept of functional organization of distributed segregated networks.

## Graphical abstract

SRTT, but not finger tapping, could induce differences in modulatory effects on the network between groups. Correlation analysis showed that faster RT was associated with higher BOLD signal in the atDCS group, whereas faster RT was mostly associated with lower BOLD signal activity in the ctDCS group and a mixture of both associations in the sham group. The polarity of tDCS differentially modulated the adaptive plasticity of remote regions using a more demanding fMRI paradigm.

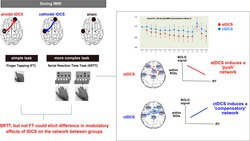

**Abbreviation**
ANOVA: analysis of variance
atDCS: anodal transcranial direct current stimulation
BOLD: blood oxygenation level‐dependent
Broadmann area: BA
CG: cingulate gyrus
ctDCS: cathodal transcranial direct current stimulation
DLPFC: dorsolateral prefrontal cortex
dPMC: lateral dorsal premotor cortex
EPI: echo planar image
FC: functional connectivity
FT: finger tapping
FWHM: full‐width half‐maximum
GABA: gamma‐aminobutyric acid
GLM: general linear model
IFG: inferior frontal gyrus
IPL: inferior parietal lobule
IPS: intraparietal sulcus
le: left
M1: primary motor cortex
MNI: Montreal Neurological Institute
PMC: lateral premotor cortex
ri: right
ROI: region of interest
RT: reaction time
SMA: supplementary motor area
SMG: supramarginal gyrus
SPL: superior parietal lobule
SRTT: serial reaction time task
tDCS: transcranial direct current stimulation

## 1 Introduction

Transcranial direct current stimulation (tDCS) has been the target of increasing interest over the last decade in relation to modulation of brain function in healthy (e.g., Reis et al. 2008; Reis et al. 2009) and clinical populations (e.g., Hummel et al. 2005; Kim et al. 2009; Zimerman et al. 2012; Stagg et al. 2012; Floel 2014; Filmer et al. 2014). TDCS has been demonstrated to induce a shift in neuronal membrane polarization, with depolarization occurring in response to anodal stimulation (atDCS) and hyperpolarization resulting from cathodal stimulation (ctDCS); therefore, anodal stimulation increases cortical excitability, whereas cathodal stimulation leads to an inhibition (Nitsche and Paulus 2000, 2001). A recent study demonstrated that atDCS enhances and ctDCS suppresses neuronal activity in the primary motor cortex (M1) (Wang et al. 2022), which is attributed to the influence of local neurotransmitter concentrations of gamma‐aminobutyric acid (GABA) and glutamate (Stagg et al. 2009). The application of atDCS to M1 has been demonstrated to result in a decline in the local GABA concentration (Stagg et al. 2009; Liebetanz et al. 2002; Kim et al. 2014), which is associated with an increase in the strengthening of functional connectivity across the resting motor network (Stagg et al. 2014; Sehm et al. 2012; Bachtiar et al. 2015). Cathodal tDCS has been shown to exert its inhibitory effect by reducing excitatory glutamatergic neurotransmission and decreasing GABA concentration (Stagg et al. 2009). This may be a contributing factor to the enhanced motor skill acquisition observed with atDCS in comparison to sham stimulation (Nakashima et al. 2021; Savic and Meier 2016; Nitsche et al. 2003; Kantak et al. 2012; Saucedo Marquez et al. 2013; Galea et al. 2011; Kang and Paik 2011; Weller et al. 2020).

The impact of atDCS and ctDCS on brain networks remains to be resolved. To date, controversial fMRI results have been obtained with atDCS and ctDCS. A grasping hand movement showed a signal increase in M1 when atDCS was applied over M1 (Jang et al. 2009; Kwon and Jang 2011). A reduced mean number of activated voxels in the supplementary motor area (SMA) was observed during the finger opposition task when ctDCS was applied over M1, although no effect was found in M1 itself. With atDCS, there was a nonsignificant increase in activated pixels with no regional differences (Baudewig et al. 2001). FMRI studies revealed no significant activation changes in anodal stimulated M1 during tapping tasks (Antal et al. 2011). FMRI activation during ctDCS also did not demonstrated any significant activation differences when compared with the sham group. Activation in the SMA was found to decrease during atDCS (Antal et al. 2011). In a resting state fMRI study utilizing anodal stimulation of M1, an increase in activation was observed in M1 and SMA on the stimulated side, as well as in the posterior parietal cortex on the non‐stimulated side (Kwon et al. 2008).

It has been argued that the fMRI‐related blood oxygenation level–dependent (BOLD) response may not be the most appropriate method for detecting the modifying capacity of tDCS (Antal et al. 2011) and that positron emission tomography may be a more effective alternative (Lang et al. 2005). However, it is important to note that the majority of the paradigms employed in previous fMRI studies utilized a simple task (e.g., finger tapping [FT]) or resting state fMRI, where the effort required to complete the fMRI condition was at a low level. This may be one reason why different fMRI studies have shown inconsistent results. There is a consensus that the BOLD response increases with the complexity of the fMRI task (Gerloff et al. 1998; Riecker et al. 2010; Foltys et al. 2003). The present study therefore investigated the influence of atDCS versus ctDCS on networks using two different fMRI paradigms with different levels of complexity. The FT task was utilized as a simple fMRI task, and an implicit serial reaction time task (SRTT) was employed as a more demanding fMRI paradigm requiring greater cognitive control (Robertson 2007). In the SRTT, subjects learn a sequential pattern of finger presses without being aware of the underlying pattern, resulting in a faster reaction time (RT). It has been discussed that the SRTT is not only a motor learning task but also involves learning sequential motor behaviour (Robertson 2007; Hardwick et al. 2013) by using perceptual learning elements (Robertson 2007).

In the present study, participants were randomly assigned to one of three groups: two real stimulation groups, one with atDCS over M1, another with ctDCS over M1, and a group that received sham stimulation. The stimulation was applied during an ongoing fMRI scan, while participants performed a simple 1‐Hz FT and the SRTT in consecutive runs. This methodology contrasts with previous fMRI studies that have examined fMRI activation after SRTT training (Nakashima et al. 2021) or between SRTT training sessions (Meehan et al. 2011) or that have used a resting state fMRI paradigm in a relatively small number of subjects, using only ctDCS and sham over M1 (Sehatpour et al. 2020).

It was suggested that there would be no significant differences in activation between the three groups when a simple motor task (FT) was performed. However, a significant difference in activation between the groups was suggested for the more demanding motor task (SRTT) when comparing atDCS with ctDCS.

Recent fMRI studies have elucidated the networks associated with SRTT. These studies have identified the involvement of several cortical and subcortical regions, including the SMA, the lateral premotor cortex (PMC), the dorsolateral prefrontal cortex (DLPFC), parietal regions and subcortical regions, and the cerebellum. These regions shared a common network during SRTT (Hikosaka et al. 2002; Grafton et al. 1995; Hazeltine et al. 1997; Grafton et al. 1998; Honda et al. 1998; Schendan et al. 2003; Hardwick et al. 2013; Tzvi et al. 2014; Baldassarre et al. 2021). These regions have been shown to encode effector‐independent learning sequences. In contrast, the primary motor cortex (M1) has been identified as an effector‐dependent area (Grafton et al. 1998). The present study was driven by the knowledge of the network associated with the SRTT, with the objective being of determining how the BOLD signal of SRTT‐related network is modified under the influence of anodal versus cathodal tDCS. The M1 was selected as the target region for stimulation on the basis of the finding that the RT of the SRTT is significantly reduced in subjects with anodal stimulation over M1, but not over PMC and DLPFC (Nitsche et al. 2003). The relationship between the BOLD signal of a specific region of interest (ROI) and RT was analyzed to investigate the effect of atDCS, compared with ctDCS, on the BOLD signal. It has been proposed that the facilitated RT of the SRTT with anodal stimulation over M1 is an effect of direct M1 stimulation. Alternatively, it has been suggested that the faster RT is based on the priming of incoming signals from other regions (Nitsche et al. 2003). The modulatory effect of tDCS was hypothesized to be evident in the more demanding fMRI paradigm of the SRTT. It was expected that with an increase in excitability resulting from atDCS would lead to a faster RT in the SRTT, which would be associated with higher BOLD signal activity. Conversely, the inhibitory effect of ctDCS was expected to result in a slower implicit time course of RT, associated with a reduced BOLD signal increase.

## 2 Methods

### 2.1 Trial Design and Participants

The study was conducted as a randomized control trial. The study comprised two intervention groups and one control group. The groups were allocated in a 1:1:1 ratio. The study population comprised 75 healthy subjects, who were randomly assigned to three groups of 25 subjects each (nine males in each group; aged between 22 and 26 years). One group received anodal tDCS (atDCS), the second group received cathodal stimulation (ctDCS), and the last group received a sham stimulation (sham). In the final analysis, two subjects in the atDCS group were excluded from final analysis (one subject had a strong head movement during the fMRI and one subject felt that the task was repeated during the SRTT). Three subjects in the ctDCS group and one in the sham group were excluded from the study due to head movement during the fMRI. Handedness was assessed using the 10‐item Edinburgh Handedness Inventory (Oldfield 1971). Participants with a laterality quotient falling outside the range of 0.5–1.0 were excluded to ensure that all subjects were right‐handed. Subjects with metal elements in or on their body (e.g., pacemakers, cochlear implants), neurological or psychiatric disease, drug or medication use, seizures, or previous brain injury were not included.

Subjects were recruited through advertisements at the university. Those who were interested in participating were subsequently informed about the study. The inclusion and exclusion criteria were then checked to determine whether the subjects met the necessary eligibility requirements. Written informed consent was obtained from all subjects. The study was conducted in accordance with the Declaration of Helsinki and was approved by the local ethics committee (No. 3485‐06/12).

### 2.2 tDCS

TDCS (NeuroConn GmbH, Ilmenau, Germany) was induced at 1 mA through saline‐soaked sponge electrodes with a surface area of 5 × 7 cm^2^, which were compatible with the MRI environment. The electrodes were placed before the subject entered the MR scanner. For the stimulation of the left primary motor cortex, the anodal stimulation electrode was placed over C3 (in accordance with the international 10‐20 system) (Nitsche et al. 2003), and the reference electrode (cathode) was placed over the contralateral orbita. Throughout the entire fMRI session, tDCS was administered in both real stimulations. In the sham group, the current was ramped up and switched off after 10 s.

### 2.3 MRI Acquisition

MRI was performed in a 3‐T whole‐body MRI system (TIM‐TRIO, Siemens, Erlangen, Germany) equipped with a standard head coil.

For fMRI, contiguous multi‐slice echo planar images (EPI; TE 30 ms) were acquired in axial orientation. Thirty‐three axial slices (3.9‐mm thickness) were acquired every 1.75 s with a voxel size of 3 × 3 × 3.9 mm^3^ in a 64 × 64 matrix with a 90° flip angle.

For T1‐weighted MRI, 192 sagittal slices (1 × 1 × 1 mm^3^ voxel size, TE 3.03 ms) were acquired in order to facilitate image co‐registration.

All visual signals of both fMRI sessions were controlled by a PC running “Presentation” software (Neurobs, Neurobehavioral‐System), which was synchronized with the scanner.

A four‐button response pad was placed under each subject's right hand prior to the start of the fMRI scan. For the visual stimulus display, subjects viewed a screen positioned in front of the scanner bore with a horizontal field of view of 19° through a prism glass.

The fMRI conditions were applied in a block design. During the REST condition, subjects were instructed to fixate a cross displayed centrally on the screen. During the active FT condition, the right index finger was tapped on the response pad at 1 Hz in response to a 1‐Hz flashing cross on the screen. Motor responses were checked by inspection within the scanner. Each condition block lasted for a duration of 30 s. FT blocks were repeated five times, alternating with REST blocks. The total scanning time for FT was approximately 5 min.

**FIGURE 1:**
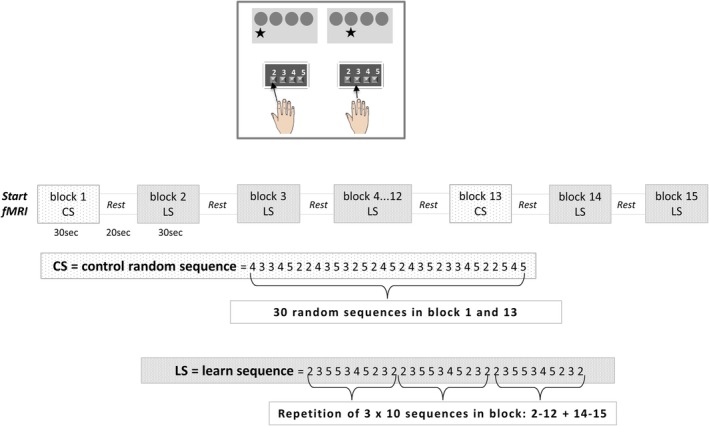
Scheme of the study design of the implicit serial reaction time task (SRTT). During the fMRI scan, subjects were presented with four circles arranged side by side on the screen. Participants were instructed to press one of the four buttons on the response pad as quickly as possible, depending on the corresponding position of the asterisk. Subjects were instructed to press the first button with their index finger when the asterisks appeared in the first circle, the second button with their middle finger for circle two, the third button with their ring finger for asterisks in circle three, and circle four corresponded to the fourth button, which was pressed with the little finger. The reaction time (RT) of each button press was recorded. The experiment comprised 15 active blocks, alternated with REST blocks. Blocks 2–12 and 14–15 were designated learning sequence blocks (LS), comprising 30 sequences (after 10 sequences, the implicitly learned sequence was repeated twice: 2 3 5 5 3 4 5 2 3 2–2 3 5 5 3 4 5 2 3 2–2 3 5 5 3 4 5 2 3 2). Blocks 1 and 13 comprised 30 random sequences (control sequences, CS).

During the active condition of the SRTT, four circles were displayed on the screen, arranged in a row, with an asterisk positioned below one of the four circles. Subjects were instructed to press one of the four keys on the response pad as quickly as possible, depending on the position of the asterisk. Subjects were instructed to press the first button with their index finger when the asterisks appeared in the first circle, the second button with their middle finger for circle two, the third button with their ring finger for asterisks in circle three, and the fourth button with their little finger for circle four. The RT of each button press was recorded. The experiment comprised 15 blocks of SRTTs alternating with REST. Blocks 2–12 and 14–15 were learning sequence blocks with the same 30 sequences (after 10 sequences, the implicitly learned sequence was repeated twice: 2 3 5 5 3 4 5 2 3 2–2 3 5 5 3 4 5 2 3 2–2 3 5 5 3 4 5 2 3 2). Subjects were instructed to press the key on the response pad as quickly as possible. Subjects were not informed of the repetitive character of the sequences. At the end of the MRI scan, subjects were asked whether they had noticed any repeated sequences. Those who did were subsequently excluded from the final analysis. Blocks 1 and 13 were designated as control random blocks: The sequence followed a pseudo‐random order to ensure the absence of repeated sequences (see Figure 1). Each asterisk was maintained for a period of 1 s, after which the next position was taken, even in the event that the key was pressed within 1 s. Each condition block lasted for a duration of 30 s. The scanning time was approximately 15 min for 15 blocks, alternating with REST.

### 2.4 Randomization and Blinding

Participants were randomly assigned to groups using sealed opaque envelopes (Doig and Simpson 2005). The tDCS device was programmed with a specific number for anodal, cathodal, and sham stimulation. The person responsible for preparing the subject for the fMRI was unaware of the subject group allocation and was blinded to which number on the tDCS device corresponded to which type of stimulation. The participants were blinded as to whether they were receiving real or sham stimulation. The data analyst was unaware of the group allocation.

### 2.5 Sample Size

The sample size for this study was based on a previous study (Nitsche et al. 2003), which demonstrated a significant interaction between the block of SRTT and DC stimulation during anodal tDCS over the primary motor cortex using an analysis of variance (ANOVA). The study comprised 20 healthy volunteers. In the present study, the stimulation was performed during the fMRI scan; therefore, weaker effects were expected due to the different environment of the MRI and possible exclusion of subjects due to motion artefacts in the fMRI. To address this, the number of participants in each stimulation group was increased and the number of SRTT blocks was increased from eight (Nitsche et al. 2003) to 15 blocks to ensure a sufficient number of subjects for statistical analysis.

### 2.6 Data Processing and Statistical Analysis

#### 2.6.1 SRTT

In the SRTT, the RT of each button press was measured. Only RTs from correct trials were included in the final analysis. The mean RT for each block and subject was subsequently calculated. All RTs that deviated by more than the 2.5 standard deviations from the individual mean RT of an individual subject were then excluded from further analysis.

Statistical calculations were performed using the IBM SPSS Statistics 29 (IBM Armonk, NY, USA).

##### 2.6.1.1 RT of Block 1 Between Groups

A normal distribution was tested with the Kolmogorov–Smirnov test. Levene's test was used to assess the equality of variance between groups. One‐way ANOVA was conducted to determine whether there were any statistically significant differences in the RTs of Block 1 (baseline) between the groups.

##### 2.6.1.2 Analysis of Accuracy

Repeated measures ANOVA was used to test for differences between groups in incorrect RTs. Group (atDCS, ctDCS, and sham groups) was set as the independent factor and the number of incorrect RTs per block as the dependent factor. Prior to ANOVA, a normal distribution was tested using the Kolmogorov–Smirnov test and Levene's test was used to assess the equality of variances between groups. The significance level was set at 0.05, corrected for multiple comparisons using the Bonferroni correction. Post hoc analyses were performed for significant main effects and interactions.

The mean error rate was calculated for each block. The definition of “high accuracy” was a mean error rate of less than 5% for the learning sequence blocks (Blocks 2–12 and 14–15).

##### 2.6.1.3 Awareness of Implicit Motor Learning

Subjects were asked whether they were aware of the repetition of the sequences after the MRI scan, using a standardized questionnaire to exclude those who were aware of implicit motor learning (Question about repetition of sequences: 1. Yes, there was; 2. I am not sure and 3. I am sure there was no repetition). Subjects were excluded from the study, if they reported knowledge of the repeated finger sequences, even if they were unable to specify the finger sequence accurately (exclusion for Items 1 and 2 of the questionnaire).

##### 2.6.1.4 Repeated Measures ANOVA

Repeated measures of ANOVA was used to test for differences in the repeated measures of RTs between groups. To assess changes in RT between the blocks, the ratio of RTs from Blocks 2–15 relative to Block 1 was calculated using the following formula: RT_Ratio_ (block_n + 1_) = RT (block_n + 1_)/RT (block_1_). The independent factor was group (atDCS, ctDCS, and sham groups), and the dependent factor was RT_Ratio_ (block_n + 1_). The significance level was set at 0.05, corrected for multiple comparisons using the Bonferroni correction. Post hoc analyses were performed for significant main effects and interactions.

For the blockwise comparison of the RT_Ratio_ between groups, the Student's *t*‐test was used for each block between groups if the normal distribution was given; otherwise, the Wilcoxon test was used. The significance level was set at 0.05, corrected for multiple comparisons using the Bonferroni correction.

#### 2.6.2 Functional MRI

FMRI data preprocessing and analysis were performed with SPM12 (Welcome Department of Cognitive Neurology, London, UK) running under Matlab 2017b (Mathworks, Sherborn, MA, USA). Both sessions started with REST. Online motion and distortion‐corrected fMRI volumes were calculated at the scanner (Zaitsev et al. 2006). All volumes were realigned to the first volume. Residual motion effects were eliminated by regressing the time course of each voxel on a periodic function of the estimated movement parameters. The resulting volumes were spatially normalized to a symmetric template based on the Montreal Neurological Institute reference brain using the normalization parameters estimated during segmentation of the T1‐anatomical scan. Low‐frequency components of the fMRI time series were removed by high‐pass filtering. The normalized fMRI images were then smoothed with an isotropic 8‐mm full‐width half‐maximum (FWHM) Gaussian kernel to allow valid statistical inference, according to the Gaussian random field theory (Friston et al. 2003).

##### 2.6.2.1 fMRI Statistical Analysis

The first‐level analysis was a general linear model (GLM) based on a model of the stimulation time course and the hemodynamic response function. Block onsets were convolved with a canonical hemodynamic response function. Following estimation of the GLM using the method of maximal likelihood, statistical parametric maps were generated for each of the blocks. For the second‐level analysis, a GLM with random effects was used by calculating a flexible factorial design, as implemented in SPM12, with the within‐subject factor Block (Blocks 1–15 for SRTT; Blocks 1–5 for FT) and the between‐group factor stimulation (atDCS, ctDCS, sham).

A significance threshold of *p* < 0.05 (corrected across the whole brain, family‐wise error, FWE) was used for all statistical analyses. The SPM Anatomy Toolbox v.3.0 (Eickhoff et al. 2006; Eickhoff et al. 2007; Eickhoff et al. 2005) was used for anatomical localization of all reported peak voxels, assigned to the most probable brain areas.

- To identify differences between the groups, the main effects of FT were compared between the groups (atCDS > ctDCS, atDCS > sham, etc.).
- A conjunction analysis was performed on the main effects of the SRTT of each group to show the common network of SRTT.
- The main effects of the SRTT were compared between groups (atCDS > ctDCS; atDCS > sham, etc.) in order to find the difference between groups. Only voxels that were maximally activated (the maximum global activity) in the contrast difference between groups were considered as a region of interest (ROI).
- The functional connectivity (FC) was calculated from the ROIs obtained from the differences between groups (see above, point 3) in order to examine interactions of selected ROIs with all other brain regions (Friston et al. 1997). The radius of the selected sphere for these ROIs was set to 8 mm. Following Friston et al. (1997) and Hamzei et al. (2016), the time series of each participant's selected ROIs were then extracted. The extracted time series of each participant's ROI was included as a regressor in a single subject GLM. This GLM was used to create a statistical parametric map of the participants' ROI time series. From this analysis, for each participant, a contrast‐image was related to each ROI (this contrast‐image includes the functional connectivity between the selected ROIs with the whole brain). A one‐sample *t*‐test was then performed for each group in order to identify common and different networks between the groups.
- Blockwise associations between RTs of SRTT and BOLD signal: We were interested in the relationship between the RTs and the associated BOLD signal from the ROIs. Therefore, a correlation analysis was performed between each subject's RT and their individual BOLD signal extracted from the ROIs for each block, starting with block 1. A correlation coefficient was calculated. The significance level was set at 0.05 using the Bonferroni correction for multiple comparisons.

## 3 Results

### 3.1 SRTT

#### 3.1.1 RT of Block 1 Between Groups

The RT of block 1 was not significantly different between groups (*F*[2,58] = 1.77; n.s.).

#### 3.1.2 Analysis of Accuracy

There was no significant main effect for incorrect‐RT of blocks (*F*[1,66] = 2.26; n.s.). The interaction between incorrect‐RT of blocks and group was also not significant (*F*[2,66] = 0.74; n.s.). For the learning sequence blocks (Blocks 2–12 and 14–15), the accuracy was high, with a mean error rate of no more than 5%. The error rate in Block 13 (the control sequence) increased up to 6.1% in the atDCS group. No subject demonstrated a performance level below 93% accuracy across all blocks.

#### 3.1.3 Awareness of Implicit Motor Learning

Following the fMRI procedure, subjects were asked regarding their awareness of the repetition of the sequences. One subject in the atDCS group indicated the task was repeated without specifying the finger sequences. This subject was subsequently excluded from further analysis.

#### 3.1.4 Repeated Measures ANOVA

There was a significant main effect for RT_Ratio_ (block_n + 1_) (*F*[14,924] = 47.30; *p* < 0.001), indicating an implicit motor learning effect across all groups.

A significant interaction was observed between RT_Ratio_ (block_n + 1_) and group (*F*[14,924] = 1.51; *p* < 0.045), indicating that RT changes varied between groups. A statistically significant difference was observed between the atDCS and ctDCS groups (*p* < 0.001) and between the atDCS and sham groups (*p* < 0.018). There was no statistical difference between the ctDCS and sham groups.

**FIGURE 2:**
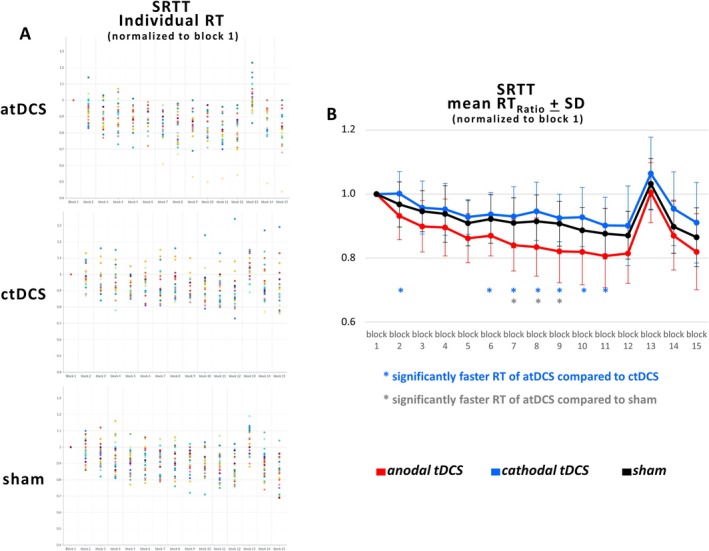
(A) The individual RTs of the anodal tDCS (atDCS), cathodal tDCS (ctDCS), and sham groups are normalized to block 1. (B) The mean RT_Ratio_ of the SRTT is presented for each group. The mean RT_Ratio_ is calculated as follows: mean RT_Ratio_ (block_n + 1_) = RT (block_n + 1_)/RT (block_1_), with block 1 designated as the control block (random sequence). Blocks 2–12, 14, and 15 were designated as sequence learning blocks, characterized by the repetition of sequences (implicitly learned sequence blocks, LS). Blocks 1 and 13 were designated as control blocks (random sequences, control sequences, CS). The mean RT_Ratio_ was then contrasted blockwise between the three groups. Blue asterisks indicate significantly faster RT between atDCS and ctDCS, while grey asterisks indicate significantly faster RT between atDCS and the sham group.

A blockwise comparison of the RT_Ratio_ between the groups revealed that the RT_Ratio_ was significantly different between the atDCS and ctDCS groups in Block 2 (*p* < 0.002), in Block 5 (*p* < 0.002), in Block 6 (*p* < 0.001), in Block 7 (*p* < 0.001), in Block 8 (*p* < 0.0002), in Block 9 (*p* < 0.0002), in Block 10 (*p* < 0.001), and in Block 11 (*p* < 0.002). The RT_Ratio_ of the atDCS group was found to be significantly different from the sham group in Blocks 7 (*p* < 0.004), 8 (*p* < 0.003), and 9 (*p* < 0.001). The RT_Ratio_ of the ctDCS group did not demonstrate a statistically significant difference compared with the sham group in any of the blocks (Figure 2).

### 3.2 fMRI

The following differences in FT were identified between the groups: A difference in fMRI activation was found between the sham and atDCS groups (sham > atDCS) in the left lateral dorsal PMC (dPMC; Montreal Neurological Institute, MNI *x*‐, *y*‐, and *z*‐coordinates: −33, −1, 59; *T* = 5.6; FWE *p* < 0.05). Other contrast comparisons did not exceed the significance level.

For the SRTT, the conjunction analysis of all groups (see Table 1) showed a common activation involving a network including cortical (dPMC, SMA, DLPFC, and other regions), subcortical (putamen, caudate nucleus, thalamus, and other regions), and cerebellar regions in both hemispheres, as previously described (Hikosaka et al. 2002; Grafton et al. 1995; Hazeltine et al. 1997; Grafton et al. 1998; Honda et al. 1998; Schendan et al. 2003; Hardwick et al. 2013; Tzvi et al. 2014; Baldassarre et al. 2021).

**TABLE 1:** Conjunction analysis of all groups (atDCS, ctDCS, and sham groups) revealed the following regions with the MNI *x*‐, *y*‐, and *z*‐coordinates.

Region	Side	*x*	*y*	*z*	*T*‐value	
Precentral gyrus	le	−27	−7	56	39.86	Area 6d3, 6d1, m6d2
Precentral gyrus	le	−36	−25	53	39.08	Area 4p, 4a
Precentral gyrus	le	−36	−13	56	37.78	Area 6d1
Precentral gyrus	ri	27	−4	53	27.67	Area 6d3, 6d1, 6d2
Postcentral gyrus	le	−54	−22	44	28.36	Area2, Area1
Postcentral gyrus	ri	42	−31	44	22.62	Area 2
SPL	le	−27	−58	59	30.67	Area 7A (SPL), Area hIP3 (IPS)
SPL	le	−33	−49	53	30.30	Area 7Pc (SPL), Area hIP3 (IPS), Area 7A (SPL)
SPL	ri	21	−64	59	25.05	Area 7A (SPL), Area 7Pc (SPL), Area hIP3 (IPS)
SPL	ri	30	−43	44	22.83	Area 7Pc (SPL), Area hIP3 (IPS)
IPL	ri	45	−58	11	15.89	Area PGp (IPL); middle temporal gyrus
IPL	ri	63	−37	20	13.09	Area PF (IPL), Area hIP3 (IPS), SMG
SMA	le	−6	−4	62	30.34	Area 6mc
preSMA	le	−3	−1	59	29.97	Area 6mr (preSMA), Area 6mc
BA 44	le	−51	2	38	28.69	IFG
Cerebellum	ri	21	−52	−22	32.81	
Cerebellum	ri	6	−64	−16	26.91	
Cerebellum	ri	9	−73	−19	26.07	

Abbreviations: BA, Broadmann area; IFG, inferior frontal gyrus; IPL, inferior parietal lobule; IPS, intraparietal sulcus; le, left; ri, right; SMA, supplementary motor area; SMG, supramarginal gyrus; SPL, superior parietal lobule.

**FIGURE 3:**
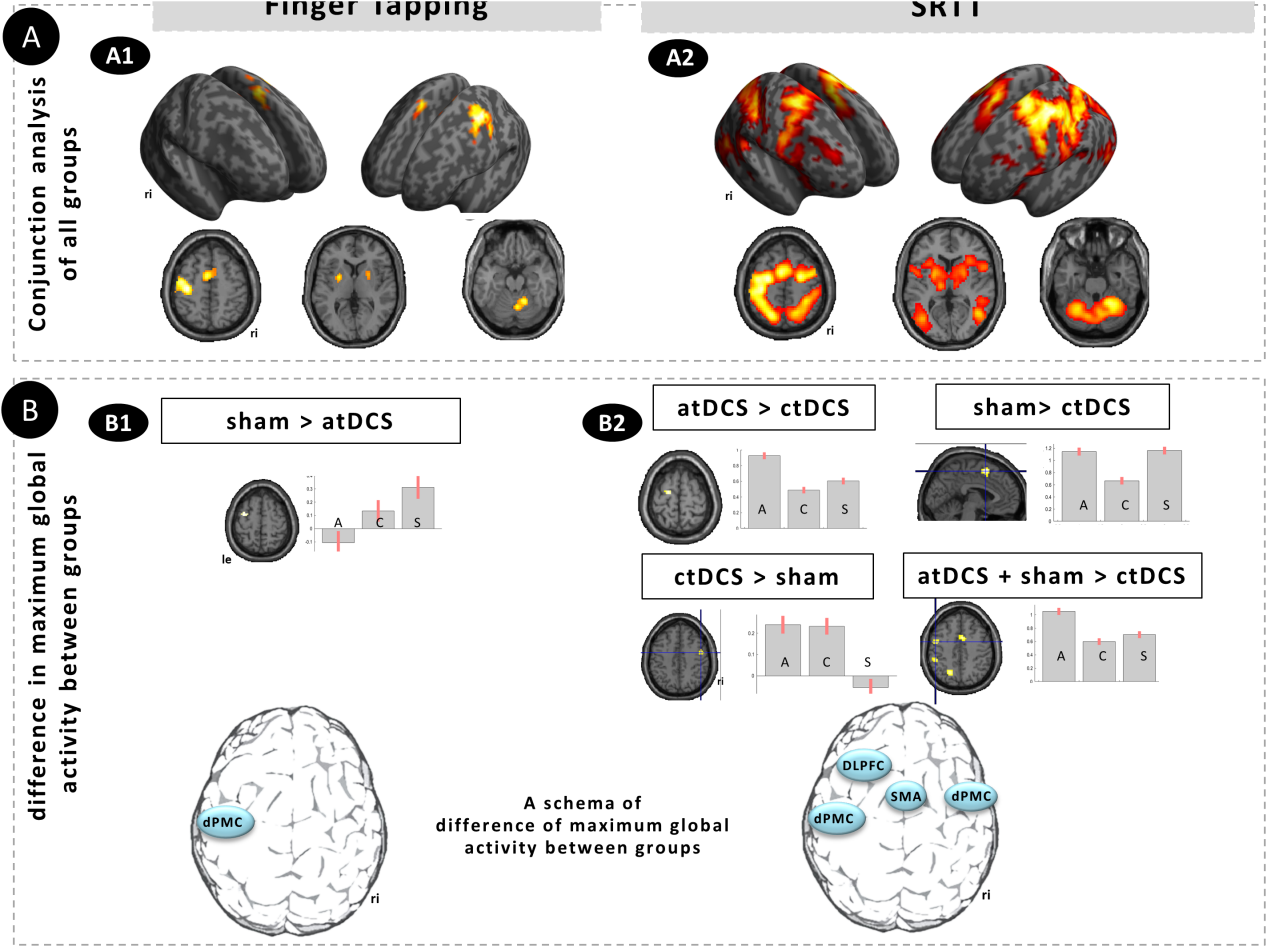
(A) A common fMRI activation was identified in all three groups in the conjunction analysis of FT (finger tapping) (A1) and SRTT (implicit serial reaction time task) (A2). (B) Differences in maximum global activity for the FT paradigm was evident in the left dPMC between the sham and atDCS group (B1). For the SRTT (B2), the analysis of the maximum global activity identified four regions: The left dorsolateral prefrontal cortex (DLPFC) exhibited a prominent activation difference in the contrast atDCS > ctDCS. Furthermore, a significant activation difference was observed in the supplementary motor area (SMA) when comparing the sham versus ctDCS group. Anodal tDCS and sham versus ctDCS showed the maximum difference in activation in the left dorsal premotor cortex (dPMC). Cathodal tDCS versus sham elicited the maximum activation difference in the right dPMC. The bars represent the activation maximum of the atDCS (A), ctDCS (C), and sham (S) groups.

In the flexible factorial design analysis for the SRTT, the maximum global activity between groups was found in four regions. These ROIs were the left DLPFC when comparing atDCS > ctDCS groups (MNI *x*‐, *y*‐, and *z*‐coordinates: −18, −7, 68; *T* = 11.83) and the left dPMC (−45, −1, 47; *T* = 8.38) when comparing the atDCS and sham groups versus the ctDCS group. The right dPMC was identified as the main difference ROI when comparing the ctDCS > sham groups (48, −4, 44; *T* = 8.39). The SMA (−3, 8, 65; *T* = 9.27) was the maximally activated ROI when comparing the sham > ctDCS groups. The following ROIs were selected for the functional connectivity analysis (Table 2; Figure 3).

**TABLE 2:** The maximum global activity of SRTT between groups showed the following MNI coordinates.

Contrast	Region	Side	*x*	*y*	*z*	*T*‐value
atDCS > ctDCS	DLPFC	le	−18	−7	68	11.83
atDCS + sham > ctDCS	dPMC	le	−45	−1	47	8.38
sham > ctDCS	SMA	le	−3	8	65	9.27
ctDCS > sham	dPMC	ri	48	−4	44	8.39
ctDCS > atDCS	No voxel exceeded the level of significance					
sham > atDCS	No voxel exceeded the level of significance					

Abbreviations: DLPFC, dorsolateral prefrontal cortex; dPMC, lateral dorsal premotor cortex; le, left; ri, right; SMA, supplementary motor area.

Functional connectivity analysis was performed for each of the selected ROIs (the left DLPFC; the left dPMC, the SMA; and the right dPMC). Table 3 shows the functional connectivity associated with each of the ROIs.

**TABLE 3:** The following table illustrates the functional connectivity from different ROIs (from Table 2) for the atDCS and ctDCS groups.

atDCS									
From	Side	*x*	*y*	*z*	To	Side	*x*	*y*	*z*
DLPFC	le	−18	−7	68	DLPFC	ri	15	35	32
DLPFC	le	−18	−7	68	dPMC	ri	36	−4	59
DLPFC	le	−18	−7	68	CG	ri	0	2	35
dPMC	le	−45	−1	47	SMA	ri	3	−10	7
SMA	le	−3	8	56	dPMC	ri	42	−1	59

ctDCS									
From	Side	*x*	*y*	*z*	To	Side	*x*	*y*	*z*
DLPFC	le	−18	−7	68	DLPFC	ri	15	32	32
DLPFC	le	−18	−7	68	dPMC	ri	45	8	35
dPMC	le	−45	−1	47	CG	le	−3	20	23
dPMC	le	−45	−1	47	DLPFC	ri	42	8	50
SMA	le	−3	8	56	dPMC	le	−39	2	38
dPMC	ri	48	−4	44	dPMC	le	−36	−1	41
dPMC	ri	48	−4	44	CG	ri	3	−1	38

Abbreviations: CG, cingulate gyrus; DLPFC, dorsolateral prefrontal cortex; dPMC, lateral dorsal premotor cortex; le, left; ri, right; SMA, supplementary motor area.

When categorizing the functional connectivity results, both the atDCS and the ctDCS shared common and distinct connectivity networks. The common networks of atDCS and ctDCS included the connectivity of the left DLPFC with the right DLPFC and with the right dPMC.

**FIGURE 4:**
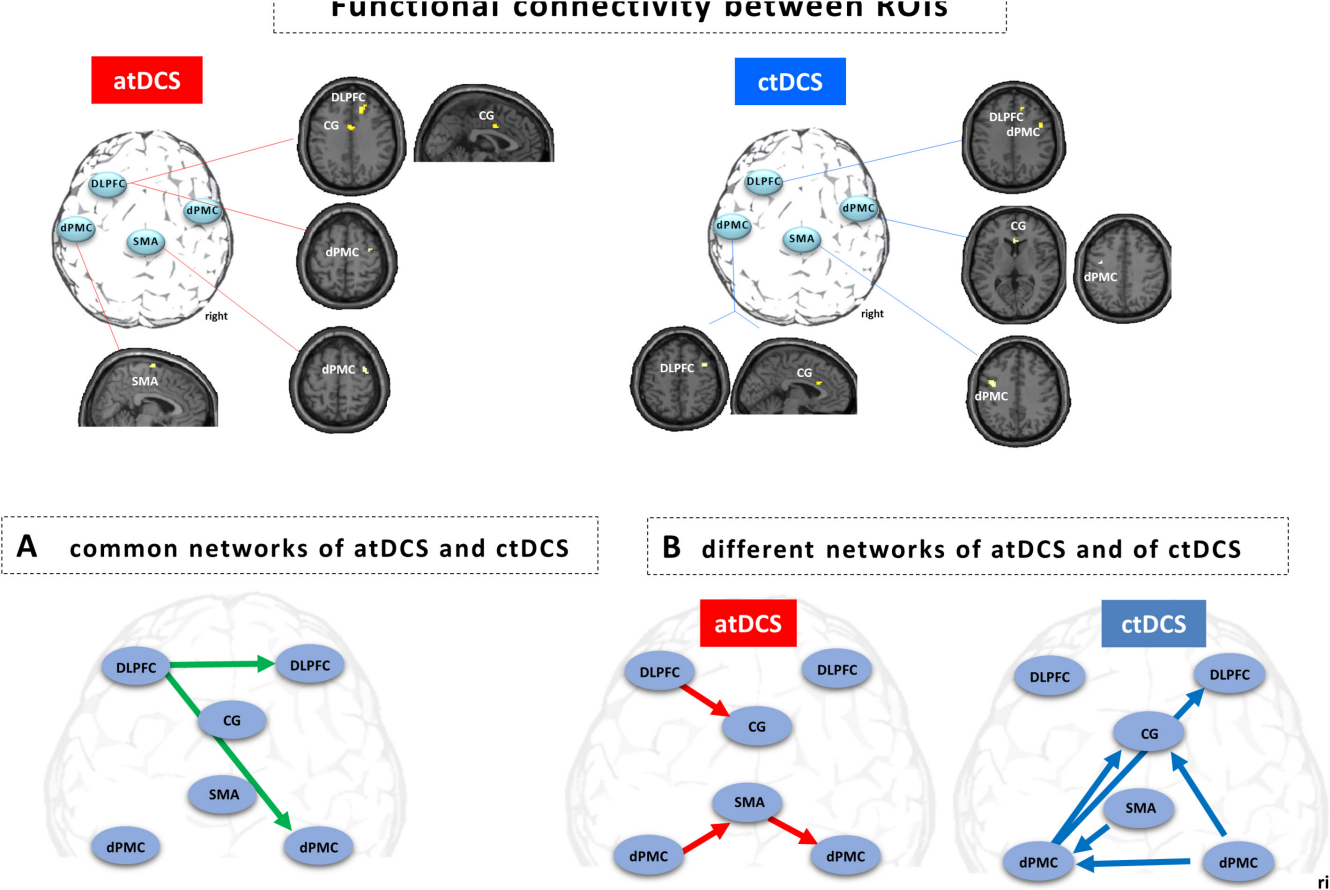
The calculation of functional connectivity was performed between regions of interest comprising left DLPFC, SMA; left dPMC; and right dPMC (see Figure 3) for each group. (A) Common functional connectivity was identified between left DLPFC and right DLPFC, as well as between left DLPFC and right dPMC in both the atDCS and ctDCS groups. (B) In addition to the common network (A), different networks were found between atDCS and ctDCS during SRTT.

Differences in functional connectivity were found for the atDCS group for the left DLPFC that was connected to the cingulate gyrus (CG) and for the left dPMC with a connection to the SMA, which was connected to the right dPMC. In contrast, the ctDCS group showed a connectivity between the left dPMC with the CG and with the right DLPFC. The right dPMC showed a connectivity with the CG and with the left dPMC. The SMA was functionally connected to the left dPMC (see Figure 4 and Table 3).

**FIGURE 5:**
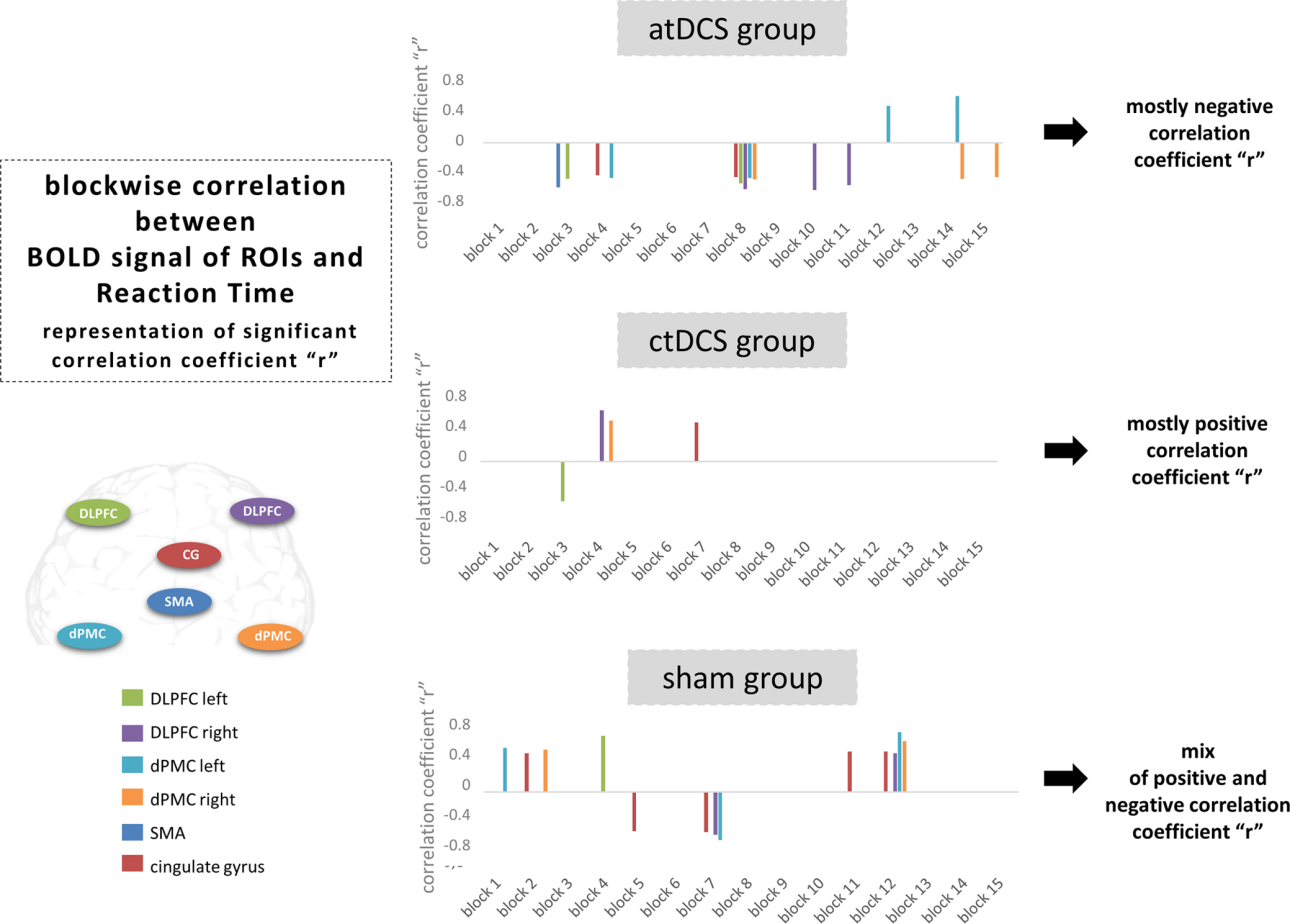
A blockwise correlation analysis was performed between the mean BOLD signal of each ROI (left DLPFC, left dPMC, right dPMC, right DLPFC, SMA, and CG) and the reaction time (RT) for each block in each group. The results of this analysis revealed significant positive and negative correlation coefficients for each block and each group over the SRTT blocks (see also Table 4). The atDCS group showed predominantly negative correlations in the ROIs (faster RTs were found to be associated with higher levels of BOLD signal activity), whereas the ctDCS group exhibited mainly positive correlations in the ROIs (lower BOLD signal activity was found to be associated with faster RTs). The sham group demonstrated a combination of positive and negative correlations (please also see Table 4).

Blockwise correlations between the RT and the BOLD signal from different ROIs that were selected on the basis of maximum global activity difference between groups (the left DLPFC; the left dPMC, the right dPMC and the SMA) and from the functional connectivity (the CG and the right DLPFC) showed differences for the atDCS and ctDCS groups. Significant correlations are shown in Figure 5 and Table 4. In the atDCS group, the majority of correlations were found to be negative, while in the ctDCS group, the majority of correlations were found to be positive. In the sham group, however, a mixture of positive and negative correlations was observed (Figure 5).

**TABLE 4:** Blockwise correlation analysis between reaction time (RT) and BOLD signal activity of different ROIs during SRTT for each group (atDCS, ctDCS, and sham groups).

atDCS	Block 1	Block 2	Block 3	Block 4	Block 5	Block 6	Block 7	Block 8	Block 9	Block 10	Block 11	Block 12	Block 13	Block 14	Block 15
SMA			−0.56												
CG				−0.41				−0.43							
le DLPFC			−0.45					−0.51							
ri DLPFC								−0.58		−0.59	−0.53				
le dPMC				−0.44				−0.44				0.46		0.58	
ri dPMC								−0.46						−0.45	−0.43

ctDCS	Block 1	Block 2	Block 3	Block 4	Block 5	Block 6	Block 7	Block 8	Block 9	Block 10	Block 11	Block 12	Block 13	Block 14	Block 15
SMA															
CG							0.49								
le DLPFC			−0.5												
ri DLPFC				0.64											
le dPMC															
ri dPMC				0.51											

sham	Block 1	Block 2	Block 3	Block 4	Block 5	Block 6	Block 7	Block 8	Block 9	Block 10	Block 11	Block 12	Block 13	Block 14	Block 15
SMA															
CG		0.44			−0.45		−0.46				0.46	0.46			
le DLPFC				0.64											
ri DLPFC							−0.49					0.44			
le dPMC	0.5						−0.55					0.68			
ri dPMC		0.48										0.58			

Significant correlations (corrected for multiple comparisons, Bonferroni correction) are shown with their correlation coefficients “*r*.”

Abbreviations: CG, cingulate gyrus; DLPFC, dorsolateral prefrontal cortex; dPMC, lateral dorsal premotor cortex; le, left; ri, right; SMA, supplementary motor area.

## 4 Discussion

Firstly, the investigation aimed to ascertain whether a more complex fMRI paradigm could provide superior insight into the differential effects of anodal and cathodal tDCS compared with a simpler fMRI paradigm. Finally, the objective was to draw novel conclusions for the SRTT network.

### 4.1 Simple Versus Complex fMRI Paradigm

The SRTT, as a more demanding fMRI paradigm, was found to elicit different modulatory influences of anodal versus cathodal tDC stimulation, in contrast to a less demanding sequential FT task. During FT, the only difference in activation was found in the left dPMC between the sham and the atDCS groups.

Using the SRTT as an fMRI paradigm, we were able to demonstrate the different effects of atDCS and ctDCS. This finding may help to explain the inconsistent results reported in previous fMRI studies employing simple tasks (e.g., FT) or resting state fMRI, which have demonstrated conflicting effects of atDCS and ctDCS.

### 4.2 Differential Impact of atDCS and ctDCS on Networks

The conjunction analysis of SRTT revealed a common network across all stimulation groups. This network comprised cortical, subcortical, and cerebellar regions in both hemispheres, as has been described previously (Hikosaka et al. 2002; Grafton et al. 1995; Hazeltine et al. 1997; Grafton et al. 1998; Honda et al. 1998; Schendan et al. 2003; Hardwick et al. 2013; Tzvi et al. 2014; Grafton et al. 1998). Consequently, during implicit motor learning, a broad network was active, irrespective of the type of stimulation, while the RTs were reduced.

The RT in the SRTT demonstrated a decline across all study groups, although the extent of decline varied. Anodal tDCS resulted in a significantly faster decrease in RT in Blocks 2 and 5–11 when compared with the ctDCS group and in Blocks 7–9 compared with the sham group (Figure 2). The question of how atDCS enables significantly faster RTs compared with the other groups, and how the reduction in RT occurred in the ctDCS group despite the fact that ctDCS suppresses neuronal activity in the primary motor cortex (M1), is of interest (Wang et al. 2022).

Analysis of the main effects (maximum global activity) was conducted among the three distinct groups (atDCS, ctDCS, and sham), which yielded BOLD signal differences mainly in four regions: the left DLPFC, the SMA, the left and the right dPMC. Subsequent to this, the functional connectivity analysis demonstrated that these four regions were functionally strongly interconnected, and with the right DLPFC and the CG, two further regions were additionally involved in the functional interaction. Despite the inherent complexity of the network, the focus of the subsequent analysis was on these regions in order to ensure clarity. In the following functional connectivity analysis, anodal and cathodal tDCS revealed both common and distinct functional interactions between the selected ROIs during the SRTT. When the correlation between RT and the BOLD signal of each ROI was investigated on a blockwise basis, the atDCS group demonstrated a negative correlation in almost all regions, while the ctDCS group exhibited mostly positive correlations. The sham group exhibited a mixture of positive and negative correlations (see Figure 5). In the atDCS group, faster RTs were associated with higher BOLD signal responses, while in the ctDCS group, faster RTs were associated with lower BOLD signal responses.

It can be hypothesized that anodal tDCS may accelerate RTs by targeting a smaller number of brain regions associated with higher levels of BOLD signal activity. We termed this association as a “push” network, involving fewer regions with high BOLD signal activity in order to accelerate RTs following a decrease in GABA concentration in M1 with atDCS over M1 (Stagg et al. 2009; Liebetanz et al. 2002; Kim et al. 2014).

**FIGURE 6:**
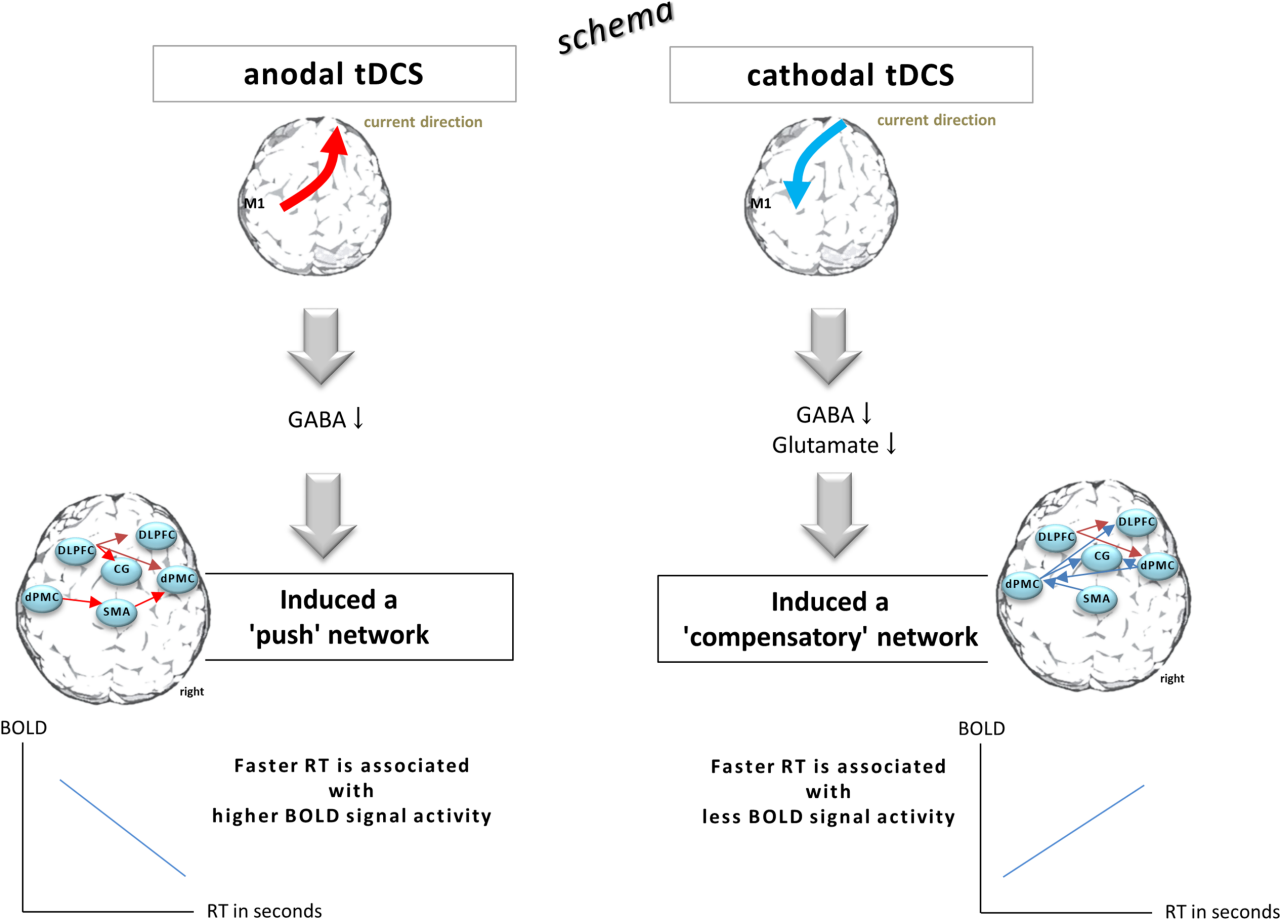
Schematic representation. The opposite current direction of atDCS and ctDCS resulted in a common and distinct network response, along with implicit motor learning of SRTT. With atDCS, a reduced number of ROIs were active to achieve an increase in RT. The BOLD signal demonstrated a predominantly negative correlation with RT. Faster RTs were found to be associated with higher levels of BOLD signal activity. This association was thus defined as the “push” network. In contrast, a greater number of ROIs were involved in the functional interaction in the ctDCS group, where RT progression was slower. Lower BOLD signal activity was observed to be associated with faster RTs. This association was defined as the “compensatory” network.

The ctDCS group also implicitly shortened their RT with less progression compared with the atDCS group. This finding is further supported by the results of the functional connectivity analysis, which revealed a greater number of regions being involved (see Figure 4). This finding suggests the presence of a compensatory mechanism that may be in response to the inhibitory effect of ctDCS (Stagg et al. 2009). Furthermore, faster RTs were associated with lower BOLD signal responses. Büchel et al. have demonstrated that a decrease in activation also reflects a learning process associated with increased efficiency and changes in connectivity between network nodes, which is clinically manifested as improved performance (Buchel et al. 1999), as seen in the ctDCS group with reduced RT. Cathodal tDCS has been demonstrated to reduce excitatory glutamatergic neurotransmission and decrease GABA concentration (Stagg et al. 2009), leading to a reduction in intracortical excitability (Nitsche and Paulus 2000). It has been suggested that with a moderate overall decrease in cortical excitability may facilitate the learning process by focusing on neurons involved in the learning process (Nitsche et al. 2003) through noise reduction (File et al. 1999). Consequently, we suggest that ctDCS may have resulted in a network response that was compensatory, characterized by involvement of more regions in the functional interactions in comparison to the atDCS group. We referred to this association as a “compensatory” network reaction (see Figure 6).

### 4.3 The DLPFC

Using fMRI, the DLPFC has been shown to be involved during SRTT (Grafton et al. 1995; Grafton et al. 1998; Nakashima et al. 2021; Turk‐Browne et al. 2010; Meehan et al. 2011; Yang and Li 2012). The DLPFC has been implicated in several functional roles in implicit motor learning. It is assumed to maintain task‐related specification during task performance and to inhibit irrelevant information in order to enhance task‐relevant information processing (Prutean et al. 2021). It has been demonstrated that the DLPFC is involved in supporting the attentional and motor planning demands of implicit sequence learning tasks (Rose et al. 2002; Schendan et al. 2003). The DLPFC has been suggested to establish motor plans during motor sequence learning and is associated with the processing of rectified visual and proprioceptive sensory information to initiate corrective action planning in the early phase of learning (Meehan et al. 2011). A hemisphere‐specific functional role for the DLPFC has also been discussed. The right DLPFC has been suggested to be more involved in implicit working memory in learners (Yang and Li 2012), and the left DLPFC has been implicated as a neural correlate of the central executive function of working memory (Baddeley 2003).

The present study revealed that both the atDCS and ctDCS groups exhibited shared functional connectivity involving an interaction between the left DLPFC and the right DLPFC, as well as between the left DLPFC and the right dPMC. Consequently, the present study confirms that both DLPFCs are active during SRTT. However, further analysis also revealed that the DLPFC exhibited divergent functional connectivity between the atDCS and ctDCS groups (see Figure 4). In the atDCS group, the left DLPFC showed functional connectivity with the CG, while in the ctDCS group, the right DLPFC exhibited functional modulation from the left dPMC. It can thus be concluded that in order to reduce the RT during SRTT, the inhibitory effect of ctDCS requires an additional connection to the right DLPFC (from the left dPMC) to regulate corrective action planning (Meehan et al. 2011), implicit working memory (Yang and Li 2012), and cognitive control (Weller et al. 2020). This illustrates that the DLPFC needs to operate in both hemispheres during SRTT.

Recent studies have used the inhibitory and excitatory effects of tDCS to investigate the role of the DLPFC in implicit motor learning, yielding conflicting results. In some studies, anodal stimulation of the DLPFC did not change RTs (Savic et al. 2017) (Nitsche et al. 2003) (Savic et al. 2017) (Savic et al. 2019) (Liew et al. 2018). Conversely, other SRTT studies have demonstrated that anodal tDCS over the DLPFC significantly reduces RT compared with sham (Nakashima et al. 2021). A potential explanation for the divergent RT outcomes reported in previous studies may reside in the variation in current intensities and stimulation durations employed. In the study by Nitsche et al. (2003), 1 mA was utilized for a duration of 15 min, whereas Savic et al. (2017) employed 1 mA for a duration of 30 min. Savic et al. (2019) used high‐definition tDCS to stimulate at 2 mA for 20 min, while Nakashima et al. (2021) employed 2 mA for a duration of 20 min. The timing of stimulation also differed between studies (before, after, or during implicit sequence learning). It has been demonstrated that cortical regions respond differently to tDCS (Radman et al. 2009), to current intensity (Lerner et al. 2021), and to current duration (Monte‐Silva et al. 2013). Therefore, it can be hypothesized that different application approaches will have different effects on learning (Weller et al. 2020). In addition to the anodal stimulation of the DLPFC, the effect of inhibitory stimulation has also been investigated in a number of studies. It was concluded that inhibition of the DLPFC does not modulate implicit sequence learning, as no stimulatory effect was observed (Prutean et al. 2021) (Vekony et al. 2021). The present study extends this perspective by demonstrating that with the inhibitory effect of ctDCS, the network responded by recruiting more regions to create a compensatory network that reduced the RT. A recent fMRI study also supported the compensatory recruitment of regions. In this study, eight healthy subjects received cathodal tDCS over the visual cortex. The enhancement in RT in the SRTT was associated with a network response rather than a local modulatory effect (Sehatpour et al. 2020). Consequently, future studies should consider that when modulating one region, a response is expected from the connected regions. Stimulation or inhibition of one region has consequences for the entire connected network (Bestmann et al. 2005; Bestmann et al. 2008; Sehatpour et al. 2020; Leaver et al. 2022; Lappchen et al. 2015).

### 4.4 The dPMC and SMA

The left dPMC has been shown to be active during sequence acquisition and visuomotor control of movement, whereas the right dPMC is involved in learning the perceptual components of SRTT and in sequence storage (Hardwick et al. 2013). Anodal stimulation of the right dPMC has been demonstrated to enhance the RT during SRTT (Kantak et al. 2012). Conversely, anodal tDCS over the dPMC has not been observed to modulate motor performance in another study (Nitsche et al. 2003). Anodal tDCS over the left dPMC during rapid eye movement sleep has been shown to improve the RT of the SRTT, thereby emphasizing the role of the left dPMC in motor memory consolidation (Nitsche et al. 2010). The SMA has been suggested to function as a sequence encoding area (Grafton et al. 1998) (Grafton et al. 2002), and its influence on task duration and accuracy has been demonstrated (Kim and Shin 2014). Additionally, the SMA has been shown to affect the RT of implicit motor learning (Yang and Li 2012). The SMA volume has been demonstrated to be closely related to implicit learning ability (Exner et al. 2002).

The present study found that the dPMC of both hemispheres was involved during the SRTT, as shown in recent studies (Hardwick et al. 2013) (Meehan et al. 2011). The present study further demonstrated that the functional connection between the left DLPPFC and the right dPMC was exhibited in both the atDCS and ctDCS groups. However, the left dPMC showed divergent connectivity patterns in the atDCS and ctDCS groups. In the atDCS group, the left dPMC demonstrated functional connectivity with the SMA, and the SMA exhibited functional connectivity with the right dPMC. Conversely, in the ctDCS group, the left dPMC exhibited a functional connection with the right DLPFC and CG. Furthermore, the left dPMC was modulated by the SMA and by the right dPMC (see Figure 4).

In the present study, the RTs were slower in the ctDCS group but demonstrated an improvement during the SRTT session. It is suggested that the inhibitory character of ctDCS may have partially disrupted the ability of brain regions to interact with each other and to “push” the network, as observed in the atDCS group. Considering that the basis of implicit sequence learning is the flow of information across network regions (Meier and Cock 2010), and therefore, the functionally connected network had to compensate for the slower progression of RTs by activating additional regions. In the atDCS group, the left dPMC functionally modulated the remote regions (the SMA and right dPMC). Conversely, the left dPMC was modulated by the SMA and by the right dPMC in the ctDCS group. This finding suggests that this network may have been a compensatory response to the inhibitory effects of ctDCS. A similar network response has previously been described in stroke patients during recovery, where the network upregulated activity in nearby and distantly connected regions, including perilesional tissue and medial and lateral premotor cortex in both hemispheres (e.g., Ward et al. 2003a, 2003b; Ward et al. 2006; Johansen‐Berg et al. 2002; Weiller et al. 1993; Weiller et al. 1992; Newton et al. 2006; Stinear et al. 2007; Tscherpel et al. 2020; Meehan et al. 2011). This response is based on brain function that is organized into segregated yet integrative networks involving both hemispheres. In this form of functional brain organization, deficits caused by damage (e.g., stroke) or inhibitory influences (e.g., ctDCS) are partially or fully compensated within the network.

As demonstrated in previous studies, dPMC has been shown to be involved in the process of motor consolidation (Focke et al. 2017; Kantak et al. 2012; Nitsche et al. 2010). The present study confirms that both dPMCs are active during the SRTT and further support previous reports that both dPMCs are involved in the early motor consolidation process (Focke et al. 2017).

### 4.5 The Primary Motor Cortex (M1)

In recent years, tDCS has been utilized in the investigation of the role of M1 during an implicit motor sequence task (Pascual‐Leone et al. 1994; Doyon et al. 1997; Honda et al. 1998). Consistent with previous studies, the present findings show that atDCS applied to M1 resulted in a decrease in RT during SRTT (Nitsche et al. 2003; Schambra et al. 2011; Kantak et al. 2012) and that the progression of the RT increase was delayed by ctDCS of M1. The present fMRI analysis suggests that atDCS of M1 enhanced the SRTT network by boosting the network (the “push” network), while ctDCS of M1 activated additional networks to compensate for the inhibitory effect of ctDCS (the “compensatory” network). It is noteworthy that even at faster RTs, there was no difference in M1 activation between the atDCS, ctDCS, and sham groups. This finding lends further support to the hypothesis that anodal stimulation over M1 primes incoming signals from other regions (Nitsche et al. 2003). Furthermore, the present findings extend previous suggestions regarding cathodal stimulation of M1 by demonstrating that ctDCS‐induced RT acceleration is modulated by remote connected regions, rather than by the direct effect of the stimulated area. This perspective is supported by the close interactive information transfer between M1 and other regions. For example, a recent study showed that anodal tDCS of dPMC increases M1 excitability via corticocortical connections (Boros et al. 2008). Anodal tDCS over M1 has been shown to decrease the local GABA concentration (Stagg et al. 2009) (Liebetanz et al. 2002), which is associated with increased strengthening of functional connectivity across the resting motor network (Stagg et al. 2014) (Sehm et al. 2012) (Bachtiar et al. 2015). Consequently, the RT decreased more rapidly under a “pushed” network. In contrast, under the inhibitory effect of ctDCS (Stagg et al. 2009), the network was compensated by increasing the functional interaction in several regions to reduce the RT. This finding indicates that the main effect of tDCS on M1 is not merely a consequence of local M1 modulation, but rather is attributable to the modulation of an interacting network that is contingent upon the polarity of the current stimulation.

It is important to note that other fMRI paradigms may yield different results. Consequently, further research is required before a generalized statement can be made.

### 4.6 Limitations

The present study focused only on the differences in the global maximum activity between the groups. The SRTT network involves other areas, such as the hippocampus, caudate nucleus, temporal lobe regions, thalamus, and cerebellum (Poldrack et al. 2001; Gheysen et al. 2011; Mizuguchi et al. 2019; Schendan et al. 2003; Yang and Li 2012; Hardwick et al. 2013; Hikosaka et al. 2002; Nakashima et al. 2021; Baldassarre et al. 2021; Keele et al. 2003; Janacsek et al. 2020). However, these areas have not been considered in this study in order to maintain a relative overview. It is expected that there will be much higher complexity in connected regions.

In this block designed fMRI paradigm, the correlation between each subject's average RT and their average BOLD signal within the ROI in a block was investigated. In an event‐related fMRI design, each event‐related BOLD signal response can be correlated with each individual RT, which would be more sensitive and should be considered in future studies.

The FT session was of a shorter duration than that of the SRTT. Consequently, it cannot be excluded that a longer duration of the FT might have shown a greater difference in activation between the stimulation groups.

The present study employed a fixed‐dose of tDCS, and did not consider an individualized dose‐controlled tDCS application to reduce the variability of electric field intensities. It is therefore hypothesized that a different result might be obtained by eliminating the variance in electric field intensities.

In this study, we did not randomize FT and SRTT, so we cannot rule out the possibility that 1 Hz FT before SRTT may have a different influence on the stimulation groups in SRTT. This is a factor that must be taken into account in future studies.

In order to investigate the distinct impacts of atDCS and ctDCS on networks, we employed SRTT as the fMRI paradigm. It is important to note that different fMRI paradigms may give different results.

The generalizability of the results of the present study may be limited by the use of a specific stimulation protocol. It is important to note that different stimulation approaches may produce varying results. For example, anodal tDCS for 26 min (Monte‐Silva et al. 2013) and 2 mA cathodal stimulation for 13 min (Shilo and Lavidor 2019) demonstrated an inverse effect on cortical excitability. Consequently, determining the appropriate current intensity and stimulation duration is critical in ascertaining whether stimulation of a particular region will result in a faster RT (Dedoncker et al. 2016). This finding is further supported by the observation that varying doses of tDCS exert distinct effects on cognitive function (Nikolin et al. 2018) (Weller et al. 2020).

In this study, the left M1 was stimulated, and it is important to consider that specific regions within a network play a specific role in triggering learning and motor memory function (Saucedo Marquez et al. 2013; Muellbacher et al. 2002; Shibasaki et al. 1993; Nachev et al. 2008; Focke et al. 2017). For example, a comparison between repetitive transcranial magnetic stimulation of M1 and SMA revealed divergent effects on implicit motor learning. While both stimulated regions showed comparable RTs and cortical excitability (recruitment curve), the task duration was significantly reduced under stimulation of the SMA compared with the M1 (Kim and Shin 2014). RTs of the SRTT improved more with atDCS over M1 than with atDCS over dPMC (Kantak et al. 2012) (Savic and Meier 2016). Therefore, a more comprehensive understanding of the specific role of connected regions within a network for implicit motor skill learning is needed. Furthermore, it is important to expand our understanding of the extent to which specific regions are influenced by which current strength, duration (Nikolin et al. 2018) and stage of learning time (Rivera‐Urbina et al. 2022).

## 5 Summary

Using a more sophisticated fMRI paradigm, we were able to demonstrate the influence of atDCS and ctDCS on cortical brain organization. Common and distinct SRTT networks were modulated by atDCS and ctDCS to accelerate the RT. With atDCS, the SRTT network was “pushed” through the association of faster RT with higher BOLD signal activity. Conversely, ctDCS, characterized by its inhibitory character, led to the activation of additional regions to compensate for the slower RT progression.

The modulation of transcranial direct current stimulation is based on the concept of functional organization of distributed segregated networks.

## Author Contributions

F.H. designed the study, completed the study protocol, interpreted the results, and wrote the manuscript. A.R. carried out the MRI scans and analyzed MRI data. D.G. wrote the manuscript.

## Conflicts of Interest

The authors declare no conflicts of interest.

### Peer Review

The peer review history for this article is available at https://www.webofscience.com/api/gateway/wos/peer‐review/10.1111/ejn.70053.

## Data Availability

We will provide our data from current study. If there is any interest, please contact farsin.hamzei@moritz-klinik.de.
